# Terahertz Modulator based on Metamaterials integrated with Metal-Semiconductor-Metal Varactors

**DOI:** 10.1038/srep26452

**Published:** 2016-05-19

**Authors:** Muhammad Tayyab Nouman, Hyun-Woong Kim, Jeong Min Woo, Ji Hyun Hwang, Dongju Kim, Jae-Hyung Jang

**Affiliations:** 1 School of Electrical Engineering and Computer Science, Gwangju Institute of Science and Technology, 1 Oryongdong Buk-gu, Gwangju 500-712, South Korea

## Abstract

The terahertz (THz) band of the electromagnetic spectrum, with frequencies ranging from 300 GHz to 3 THz, has attracted wide interest in recent years owing to its potential applications in numerous areas. Significant progress has been made toward the development of devices capable of actively controlling terahertz waves; nonetheless, further advances in device functionality are necessary for employment of these devices in practical terahertz systems. Here, we demonstrate a low voltage, sharp switching terahertz modulator device based on metamaterials integrated with metal semiconductor metal (MSM) varactors, fabricated on an AlGaAs/InGaAs based heterostructure. By varying the applied voltage to the MSM-varactor located at the center of split ring resonator (SRR), the resonance frequency of the SRR-based metamaterial is altered. Upon varying the bias voltage from 0 V to 3 V, the resonance frequency exhibits a transition from 0.52 THz to 0.56 THz, resulting in a modulation depth of 45 percent with an insertion loss of 4.3 dB at 0.58 THz. This work demonstrates a new approach for realizing active terahertz devices with improved functionalities.

The terahertz (THz) band of the electromagnetic spectrum, with frequencies ranging from 300 GHz to 3 THz, has attracted much attention owing to its applications in a wide variety of fields ranging from spectroscopy and imaging to information and communication technology^1^. In order to realize practical terahertz systems, components and devices which can efficiently control and guide the terahertz waves, are required. This has led to demonstrations of various terahertz devices such as filters, absorbers, polarization convertors, and modulators^2^,^3^. Modulators or switches that actively manipulate terahertz waves^4^ have been used in various applications such as coded apertures for terahertz imaging^5^, carrier modulators in communication systems^6^, and terahertz optical devices^7^. Modulator devices with various modulation mechanisms such as mechanical^8^, optical^9^, electronic^10^, and thermal^11^ mechanisms have been reported. Among these, electrically controlled modulators have gathered the biggest attention and various device designs have been proposed. Graphene based electro-absorption modulators with low insertion loss and large bandwidth have been demonstrated^12^. However, the achievable modulation depth in these devices is limited due to the characteristics of graphene. Devices with improved modulation depth but narrow bandwidth have been realized by enhancing the electric field amplitude in the graphene using frequency selective surfaces or electromagnetic cavities^13^,^14^,^15^,^16^,^17^. One of the shortcomings of these devices is their low modulation speeds of only several kilohertz (kHz) owing to their large active area. High speed modulation has been demonstrated in devices based on modifying the resonant metamaterial response using substrates with reconfigurable properties. Initial device designs using this approach consist of a split ring resonator (SRR) based metamaterial on top of a doped GaAs that functions as a Schottky contact^4^,^10^,^18^. Without an applied bias, the doped GaAs in the split gaps acts as a resistance, shunting the split gap capacitance and resulting in an extremely weak resonance. Upon application of applied bias, the charge carriers in the split gap are depleted, and the split gap capacitance is no longer shunted, producing a stronger resonant response. Modulation speeds of several megahertz (MHz) have been demonstrated by such devices, which vary the charge carrier concentration in SRR split gaps using gate controlled 2-dimensional electron gas (2DEG) in GaAs and GaN based heterostructures^19^,^20^. Despite their excellent performance in terms of modulation depth and speed, these devices possess a low resonance strength and high insertion loss in transmission, because of the inherent resistive loss.

In this study, we explore a different approach for realizing terahertz modulation based on capacitive switching of the metamaterial resonance. Such an approach was originally demonstrated at microwave frequencies by integrating lumped varactor elements at the metamaterial unit cell^21^,^22^. Integrating varactors into the metamaterial unit cell allows active variation of the capacitance and hence of the resonance frequency of the metamaterial. The shifting of the resonance frequency results in modulation of the transmission amplitude at the surrounding frequencies. Implementation of this approach at terahertz frequencies presents several challenges in terms of the integration of varactor devices and the required high cut-off frequencies for operation in the THz band. Here, we demonstrate his approach by realizing high frequency metal-semiconductor-metal (MSM) varactor diodes on top of AlGaAs/InGaAs/GaAs double heterostructure which supports 2-dimensional electron gas (2-DEG) in its quantum well^23^,^24^. The 2DEG acts as an equi-potential plane, confining the Schottky depletion zones to the region between the contact and the 2DEG^25^, leading to high capacitance for a given contact area. At low bias voltages, the device capacitance is high, determined by the series combination of the two Schottky diode capacitances. As the bias voltage increases, the 2DEG underneath the reverse biased Schottky contact is depleted, resulting in a decrease of overall varactor capacitance. The cutoff frequency of the device is limited by the device series resistance, comprised of the 2DEG sheet resistance, the metal-semiconductor interfacial resistance, and the gate metal resistance^26^. The detailed behavior of the MSM-2DEG varactor operation can be found elsewhere^27^.

## Terahertz Modulator Design

**Figure 1:**
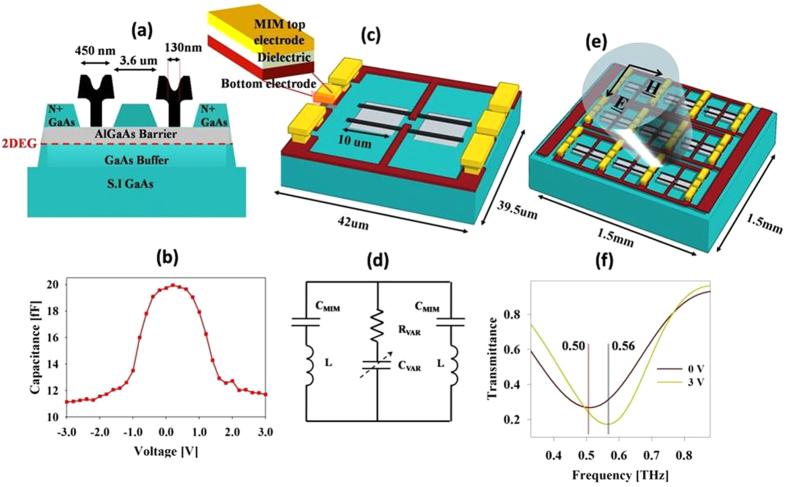
Structure and design of the THz modulator based on metamaterials integrated with metal-semiconductor-metal varactors. (**a**) Schematic of the MSM-2DEG varactor. (**b**) C-V characteristics of MSM-2DEG varactor. (**c**) Schematic view of a metamaterial unit cell. Grey areas represent the active region. The varactor gate electrodes are shown in black. The first metal layer (brown) forms split ring and the bottom electrode of the MIM capacitors while the second metal layer (yellow) forms the top electrode of the MIM capacitors. (**d**) Equivalent circuit corresponding to the metamaterial unit cell. (**e**) Schematic view of the complete device containing 1122 unit cells and the bias pads. Polarization of the incident beam is also indicted. (**f**) Simulated transmittance at the bias voltages of 0 and 3 V.

The MSM-2DEG varactors employed in this work are based on an AlGaAs/InGaAs/GaAs pseudomorphic high electron mobility (p-HEMT) structure and are designed to maximize the cutoff frequency while maintaining a sufficiently high capacitive switching ratio. The dimensions of the MSM-2DEG varactors are shown in Fig. 1(a). Each of the two T-shaped Schottky gate electrodes has a foot length of 130 nm. By reducing the contact area, the nanometer sized gate electrodes cut down the device series resistance associated with metal-semiconductor contact, while the T-shaped gate electrodes reduce the gate metal resistance component. The sheet resistance of the 2DEG between the two Schottky gates is minimized by placing the two Schottky gates at the smallest distance apart permitted by the fabrication process, which in our case is 3.6 μm. The capacitance-voltage characteristics of the fabricated varactor device are determined by using S-parameter measurements from 1 to 40 GHz with an HP8510C vector network analyzer (See methods for details). As can be seen in Fig. 1(b), owing to the symmetric geometry of the device, the MSM-2DEG varactor exhibits symmetric C-V characteristics. The maximum capacitance value of 20 fF lies at 0 V. The capacitance is approximately constant up to an applied bias voltage of 0.7 V, beyond which it exhibits a sharp decrease for applied bias voltages up to 2 V and then maintains a nearly constant value of 11.2 fF for the higher bias values. The cutoff frequency of the device, defined as^27^ f_0_ = 1/(2πR_o_C_o_), where R_o_ and C_o_ are the series resistance and the capacitance at 0 V, respectively, is determined to be 0.4 THz.

These MSM-2DEG varactors are monolithically integrated into the metamaterial based on electric split ring resonators^28^ (SRRs). The SRRs are LC resonators where the inductance L is proportional to the size of the split ring and the capacitance C is determined by the split gap. The varactors are located at the split gap, thus governing the capacitance of the SRR. The application of a bias voltage to the varactor requires that the two gate electrodes be electrically isolated which would otherwise be joined by the split ring. This constraint is satisfied by placing metal-insulator-metal (MIM) capacitors between the upper and lower halves of the split ring. Figure 1(c) schematically illustrates the metamaterial unit cell geometry and design. The device is realized via a commercial GaAs pseudomorphic high electron mobility transistors (p-HEMT) process technology. The substrate is 100-μm-thick semi-insulating GaAs, on top of which AlGaAs/InGaAs/GaAs double heterostructures are grown by using metal organic chemical deposition (MOCVD). The 130-nm-long T-gate electrodes having a gate head length of 450 nm are written using electron beam lithography. A 0.51-μm-thick metal layer connected to gate electrodes forms both the split ring and the bottom electrode of the MIM capacitors. On top of the first metal layer, layers of 150-nm-thick silicon nitride and 800-nm-thick silicon oxide are deposited, on top of which a second metal layer is deposited, forming the top electrode of the MIM capacitor. The equivalent circuit configuration corresponding to the metamaterial unit cell is depicted in Fig. 1(d). At terahertz frequencies, the high MIM capacitance acts as a low impedance path, leaving the split ring inductance unimpaired. At DC bias, the MIM capacitor impedance is large, thus achieving isolation between the two varactor electrodes.

Figure 1(f) shows the full wave electromagnetic simulation results for the metamaterial unit cell. The SRR dimensions are optimized to resonate at around 0.5 THz. The voltage dependent varactor capacitance and resistance are modeled as lumped elements in the simulation. For the maximum varactor capacitance at 0 V, the transmission resonance frequency is around 0.5 THz; this value shifts to approximately 0.56 THz for the minimum varactor capacitance at 3 V. It can also be observed that the Q-factor and the strength of the transmission resonance increase at 3 V, which is consistent with the fact that both are inversely proportional to the resonator capacitance.

Figure 1(e) provides a schematic description of the complete device. The complete device has dimensions of 1.5 × 1.5 mm^2^, containing a total of 1122 metamaterial unit cells. The vertical side bars of all the SRRs along each row are shared by the adjacent SRRs, electrically connecting all the varactor electrodes in each row. The top and bottom halves of the split rings in each row are connected to the bias pads located at the right and left perimeter of the device, thereby biasing all the unit cells in the device.

## Results and Discussion

**Figure 2:**
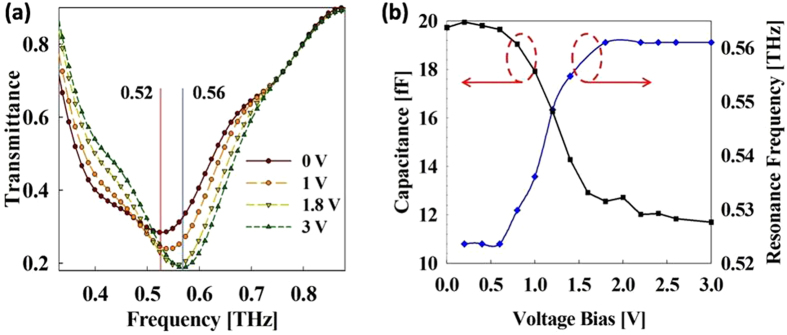
THz transmission characterization of device. (**a**) Device transmittance characteristics. (**b**) Comparison between MSM-2DEG varactor capacitance and transmittance resonance frequency.

**Figure 3:**
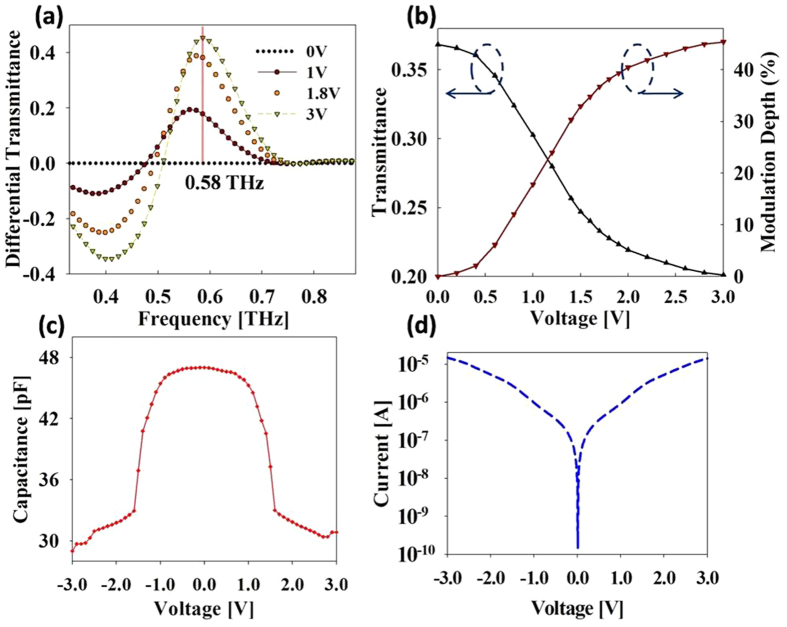
Modulator performance characterization. (**a**) Differential transmittance at various bias voltages. (**b**) Transmittance and Modulation depth as a function of voltage at 0.58 THz. (**c**) Capacitance-Voltage characteristics of the complete device containing 1122 unit cells and the bias pads. (**d**) Modulator I-V characteristics.

The fabricated device was characterized by using THz time domain spectroscopy (THz-TDS) method. The measurement was carried out at normal incidence with the electric field polarized orthogonal to the split gap to drive the metamaterial elements into resonance. The terahertz transmittance characteristics, measured under different values of applied bias, are shown in Fig 2(a). For an applied bias of 0 V, transmission resonance occurs at a frequency of 0.52 THz, which is slightly higher than that observed in the simulation. With increasing of the applied bias from 0 to 3V, the transmission resonance shifts to 0.56 THz. A comparison of the variation in the transmission resonance frequencies and the MSM-2DEG varactor capacitance for the range of applied voltage bias is presented in Fig. 2(b). As expected, the transmission resonance frequency varies in a manner similar to that of the varactor: for applied bias values of 0 to 0.7 V, the resonance frequency exhibits a very slight shift; between 0.7 and 1.8 V, a sharp transition takes place from 0.52 THz to 0.56 THz; and increasing the voltage above 1.8 V does not affect the resonance frequency in any appreciable way. For bias values above 3 V, the device transmission characteristics exhibit no variation. The experimentally measured results exhibit a fairly good qualitative agreement with the simulation results, except at higher frequencies. As can be observed in Fig. 2(a), the difference in transmittance values at different bias voltages decreases at higher frequencies and completely disappears at around 0.76 THz. This convergence of the transmittance response at higher frequencies for different values of applied voltage is a consequence of the decline in the maximum capacitance of the MSM-2DEG varactor with increasing frequency. At higher frequencies, the varactor Schottky gate electrodes and the underlying 2DEG behave as a transmission line rather than as a lumped element^29^. As the signal frequency increases, the resulting equivalent transmission line capacitance decreases and above a certain frequency, the varactor C_max_ becomes equal to the C_min_ and the varactor response becomes bias independent^27^. The lumped element varactor model employed in the simulation does not take into account this behavior and hence does not show the convergence behavior observed in the experiments. The frequency at which the C_max_ of the MSM-2DEG varactor becomes equal to its C_min_, is given by the varactor figure of merit, defined as^24^ FOM = f_0_∙C_max_/C_min_. The FOM for our varactor device comes out at 0.73 THz, which is in close agreement with the experimentally observed value of 0.76 THz. In order to characterize the variation in the transmittance amplitude arising at a given frequency, we calculate the differential transmittance *D*(*ω*), defined as *D*(*ω*) = [*T*(*ω*)_*0V*_ *−* *T*(*ω*)_*V*_]/ *T*(*ω*)_*0V*_. As shown in Fig. 3(a), *D*(*ω*) becomes larger with increasing bias voltage and has negative (positive)values below (above) the frequency at which the new transmittance curve intersects the original transmittance curve at 0 V. This behavior is a manifestation of the upward shift of the resonance frequency with increasing bias voltage, which results in a decrease (increase) of transmittance below (above) the intersection frequency. The negative (positive) values of *D(ω*) express the increase (decrease) in transmittance values with the increase in applied bias, relative to the transmittance at 0 V. The differential transmittance *D*(*ω*), calculated here, coincides with the definition of the modulation index *M.D* = [*T*_max_ − *T*_min_]/*T*_max_, as used in the other literatures, for positive values of *D*(*ω*). The maximum modulation depth, as defined above, lies at 0.58 THz, with an insertion loss, *IL* = −10 *log* [*T*_max_], of 4.3 dB. In Fig. 3(b) the device transmittance at 0.58 THz is plotted as a function of applied bias voltage. The device transmittance changes from high to low very sharply in a voltage interval of 0.7 to 1.8 V. This is in contrast to most of the previously reported terahertz modulator designs, which require large voltage swings to vary the transmission from maximum to minimum. Additionally, the small voltage levels required for the operation of the current device enable more efficient modulation of incident THz waves. The sharp change in the transmittance with the applied bias is quantitatively represented by ∆T/∆V, the total change in the transmittance with respect to the total change in applied voltage. As can be seen in Table 1, the ∆T/∆V value of 0.06 in our work is considerably larger than most previously reported values for the state of the art designs. An estimate of the device modulation speed is obtained indirectly by determining the time constant of the device. The device impedance is measured at 15 MHz using an HP4194A high frequency impedance analyzer and fitted to a series RC circuit. Figure 3(c) shows the measured capacitance of the complete device, which exhibits a voltage dependent behavior similar to that of the individual varactor. In fact, the whole device can be considered to be one large varactor with all the individual varactors connected in parallel with each other. This is apparent in the I-V characteristic curve of the complete device, shown in Fig. 3(d). The total capacitance, however, is quite large compared to the sum of all the individual varactor capacitances in the device, indicating that parasitic capacitance between unit cells and bias pads play a crucial role in determining the overall capacitance. The RC constant determined using the above values comes out to be 3.2 ns; the corresponding 3dB cut-off frequency is 48 MHz, which is higher than that value of all previously proposed modulators except ref. 20. Further improvement in modulation speed can be realized by reducing the total number of unit cells or, equivalently, the total area, thus reducing the overall capacitance. Our device has dimensions of 1.5 × 1.5 mm^2^; however for a carrier signal at 0.58 THz, a device area of 500 × 500 μm^2^, comparable to the wavelength of the carrier, will suffice. Reduction of the device area from 1.5 × 1.5 mm^2^ to 500 × 500 μm^2^ will result in an improvement of device speed to up to 0.43 GHz.

A comprehensive comparison between the current device and previously reported front line electronic terahertz modulator devices is given in Table 1. The individual performance parameters achieved in this work are smaller than the maximum values reported in previous works. However, our device represents an improved compromise between various performance parameters. Additionally, the low bias voltage and the small voltage swing, required to achieve modulation in our case, offer significant improvement in terms of efficiency and device functionality.

In conclusion, we have demonstrated an electrically controlled THz modulator device based on high speed MSM-2DEG varactors monolithically integrated into an electric split ring resonator metamaterial. The metamaterial resonance frequency was shifted from 0.52 to 0.56 THz by electrically controlling the varactor capacitance. A modulation depth of 45%, with an insertion loss of 4.3 dB at 0.58 THz, was demonstrated for a small applied voltage variation of 0–3 V. This work demonstrates a new approach for realizing active terahertz devices with improved functionality. A significant improvement in device performance can be realized by employing metamaterial designs of higher quality factor, also enabling device application as tunable terahertz filters. The proposed design can easily be scaled up to larger sizes without degrading the device performance. This property enables application of this design for realizing multi pixel spatial THz modulators required in terahertz imaging systems employing compressive sensing or single pixel detection.

## Methods

### Varactor Characterization

**Figure 4:**
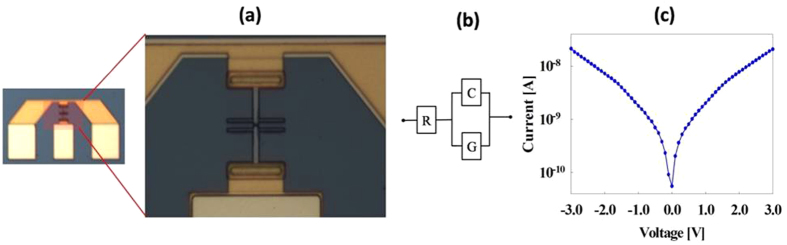
MSM-2DEG Varactor characterization. (**a**) Optical image of the MSM-2DEG varactor including bias pads. (**b**) Varactor equivalent circuit. (**c**) MSM-2DEG varactor I-V characteristics.

The Capacitance-Voltage characteristics of the MSM-2DEG varactor were determined using S-parameter measurements from 1 to 40 GHz (resolution of 1 GHz) with an HP8510C vector network analyzer. Pad parasitic elements were eliminated using a de-embedding process with an open pad pattern; the de-embedded measurements were fitted to a common varactor equivalent circuit^25^, shown in Fig. 4(b), composed of capacitance (C), series resistance (R), and parallel conductance (G). The capacitance and series resistance values were determined to give the best approximation to S-parameters throughout the above frequency range and do not include any frequency dependence. For higher frequencies, as described in the main text, the lumped element model used above is not applicable and a transmission line model of gate electrodes is required for determining the frequency dependence^27^. The DC I-V characteristics of the MSM-2DEG varactor exhibit low leakage current and are given in Fig. 4(c).

### Electromagnetic Simulation

**Figure 5:**
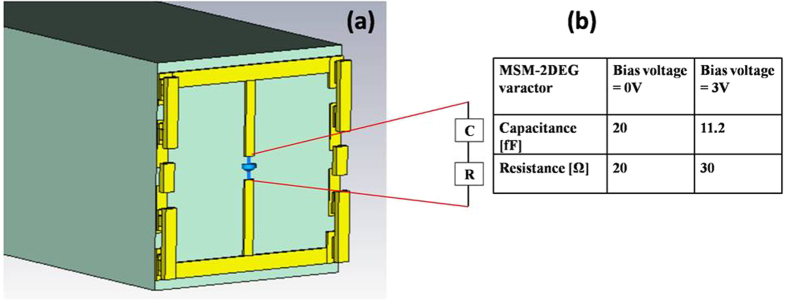
Electromagnetic simulation model of the device. (**a**) Metamaterial unit cell (dielectric layers between the two metallic layers are hidden for the sake of clarity). **(b)** MSM-2DEG Varactor values at 0 and 3 V.

Electromagnetic (EM) simulations of the metamaterial unit cell were carried out using a commercial EM simulation tool. The MSM-2DEG varactor was modeled as a lumped element consisting of series capacitance and resistance (Fig. 5), determined as described above. Periodic boundary conditions were applied to the metamaterial unit cell to mimic a practical scenario containing thousands of unit cells. A plane wave, whose polarization was orthogonal to the split gaps of the SRR structures, was used as an excitation source. To eliminate the effect of Fabry-Perrot pulses arising from the finite thickness of the substrate, simulation was carried out in the time domain and time windowing was applied to capture only the main pulse.

### Terahertz Transmission Measurement

**Figure 6:**
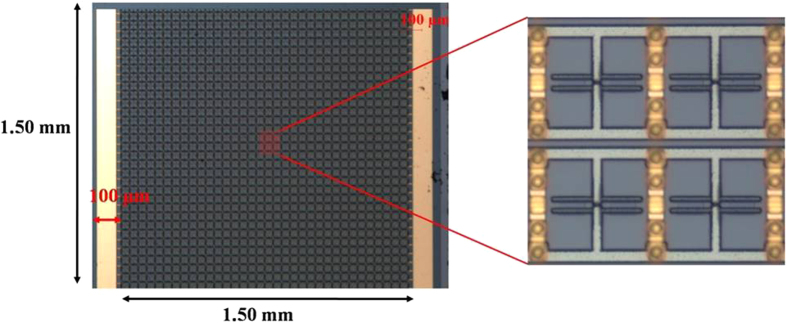


Optical images of the fabricated device are provided in Fig. 6. Terahertz transmission measurements were carried out using a conventional terahertz time domain spectroscopy setup (TPS3000) by Teraview. The fabricated device was mounted on a 500-μm-thick GaAs substrate for mechanical stability and increasing the size of time domain window of the measurement, which facilitated excluding the effect of multiple pulses due to Fabry-Perot interference. The Fourier transformed transmittance of the mounted sample was referenced by the transmittance of 500-μm-thick GaAs substrate. This operation does not remove the effect of the original 100-μm-thick substrate, but only removes the effect of additional 500-μm-thick GaAs substrate used for mounting the device. This is different from the referencing procedure done in some of the previous literatures where the effect of the substrate is totally removed by using it as the reference.

## Additional Information

**How to cite this article**: Nouman, M. T. *et al*. Terahertz Modulator based on Metamaterials integrated with Metal-Semiconductor-Metal Varactors. *Sci. Rep.* **6**, 26452; doi: 10.1038/srep26452 (2016).

## Figures and Tables

**Table 1:** Performance comparison with the previously reported electronic THz modulators.

**Performance Parameters**	**Metamaterial - GaAs Schottky contact [ref.** 10]	**Metamaterial Diffraction Grating - GaAs Schottky contact [ref.** 18]	**Graphene Electro -Absorption [ref.** 14]	**Dipole - GaN HEMT [ref.** 20]	**Metamaterial - GaAs HEMT [ref.** 19]	**Metamaterial - GaAs MSM Varactor [This work]**
Operating Frequency [THz]	0.81	0.4	0.62	0.35	0.46	0.58
Insertion Loss [dB]	5	9.4	2	3	8	4.3
Modulation Depth [%]	80	99	64	85	33	45
Operating Voltage Interval [V]	0–16	0–13	−10–20	0–7	0–3	0–3
∆T/∆V	0.015	–	0.013	0.06	0.04	0.06
Modulation Speed	30 kHz	1 kHz	4 kHz	1 GHz	10 MHz	48 MHz
